# Systemic disease presenting as cardiac tamponade: a case report

**DOI:** 10.1093/ehjcr/ytae137

**Published:** 2024-03-15

**Authors:** Maria Inês Barradas, Inês Coutinho dos Santos, Fabiana Duarte, Anabela Tavares, Dinis Martins

**Affiliations:** Department of Cardiology, Hospital do Divino Espírito Santo de Ponta Delgada, Avenida D. Manuel I, 9500-370 Ponta Delgada, São Miguel, Portugal; Department of Cardiology, Hospital do Divino Espírito Santo de Ponta Delgada, Avenida D. Manuel I, 9500-370 Ponta Delgada, São Miguel, Portugal; Department of Cardiology, Hospital do Divino Espírito Santo de Ponta Delgada, Avenida D. Manuel I, 9500-370 Ponta Delgada, São Miguel, Portugal; Department of Cardiology, Hospital do Divino Espírito Santo de Ponta Delgada, Avenida D. Manuel I, 9500-370 Ponta Delgada, São Miguel, Portugal; Department of Cardiology, Hospital do Divino Espírito Santo de Ponta Delgada, Avenida D. Manuel I, 9500-370 Ponta Delgada, São Miguel, Portugal

**Keywords:** Systemic lupus erythematosus, Cardiac tamponade, Polyserositis, Case report

## Abstract

**Background:**

Systemic lupus erythematosus (SLE) is a chronic autoimmune disease characterized by multisystem inflammation and is a common cause of pericarditis and pericardial effusion, but significant pericardial effusion and cardiac tamponade are rare and even rarer as the first manifestation.

**Case summary:**

We report the case of a young male who presented with fever, recurrent pericarditis, and polyserositis with pericardial and bilateral pleural effusion. On examination, he was haemodynamically unstable and the pericardial effusion had considerable dimensions and an urgent pericardiocentesis was performed. Antinuclear antibody with a speckled pattern was positive, complement C4 levels were low, and the remaining autoimmunity and infectious study was unremarkable. Considering the European League Against Rheumatism/American College of Rheumatology classification criteria for SLE, a score of 11 points was obtained, confirming the diagnosis of SLE.

**Discussion:**

This case report illustrates a rare form of presentation of SLE, in which the first manifestation was pericarditis with polyserositis and cardiac tamponade.

**Learning points**

- A high clinical suspicion is essential for the diagnosis of autoimmune systemic diseases.
- Although infrequent, pericardial effusion and cardiac tamponade may be the first manifestations of systemic lupus erythematosus.

## Introduction

Acute pericarditis is a frequent cause of chest pain in young adults and is frequently accompanied by pericardial effusion, often small. In most cases, no cause is identified, or a presumably viral infection is assumed. Alternative aetiologies may have treatment and prognostic implications and should be suspected in recurrent or severe cases. Systemic lupus erythematosus (SLE) is a chronic autoimmune disease characterized by multisystem inflammation and is a common cause of pericarditis and pericardial effusion, but significant pericardial effusion and cardiac tamponade are rare and even rarer as the first manifestations.^1^ The diagnosis of SLE encompasses a clinical challenge and requires a high clinical suspicion.

## Summary figure

**Figure:**
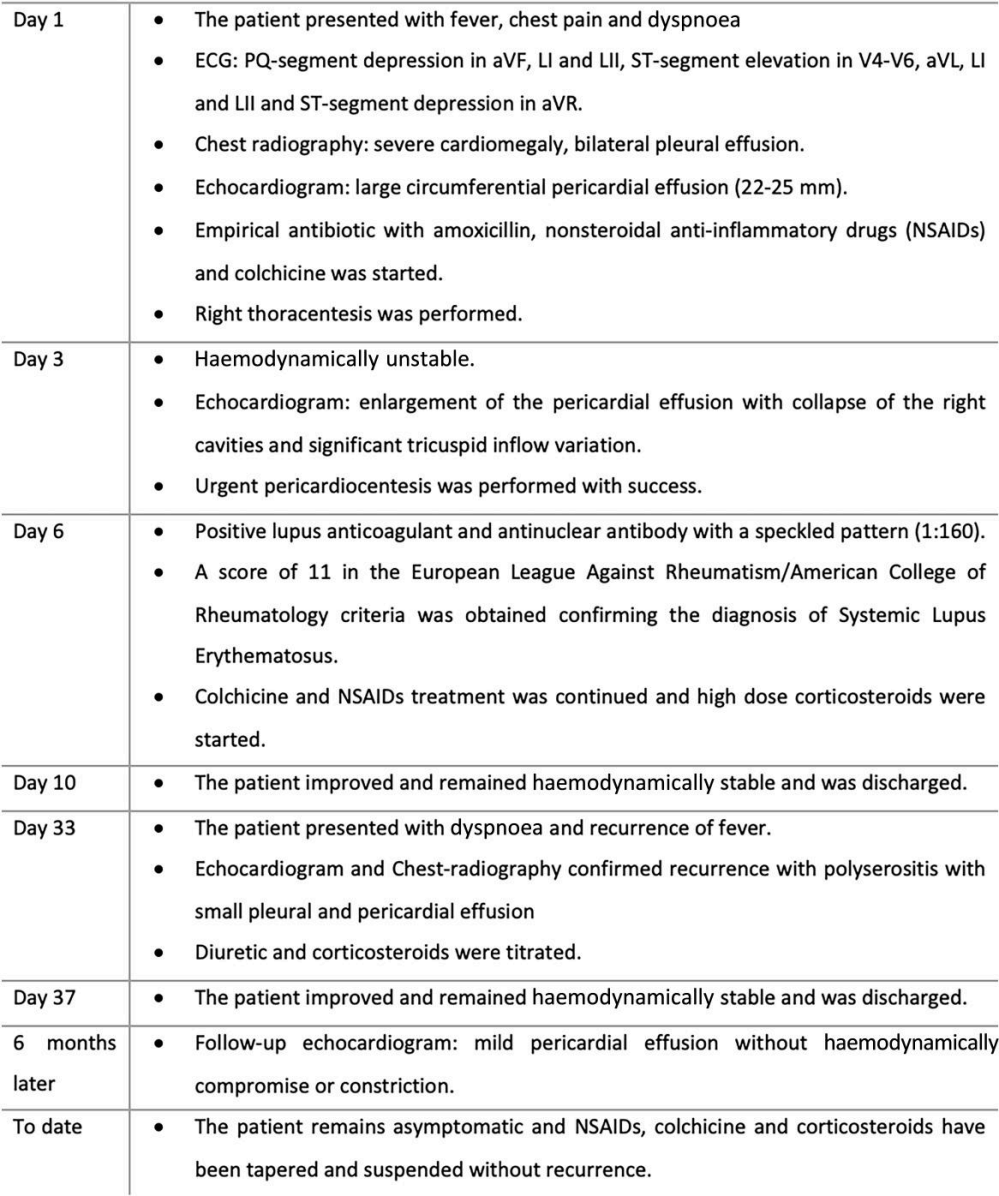


## Case presentation

**Figure 1:**
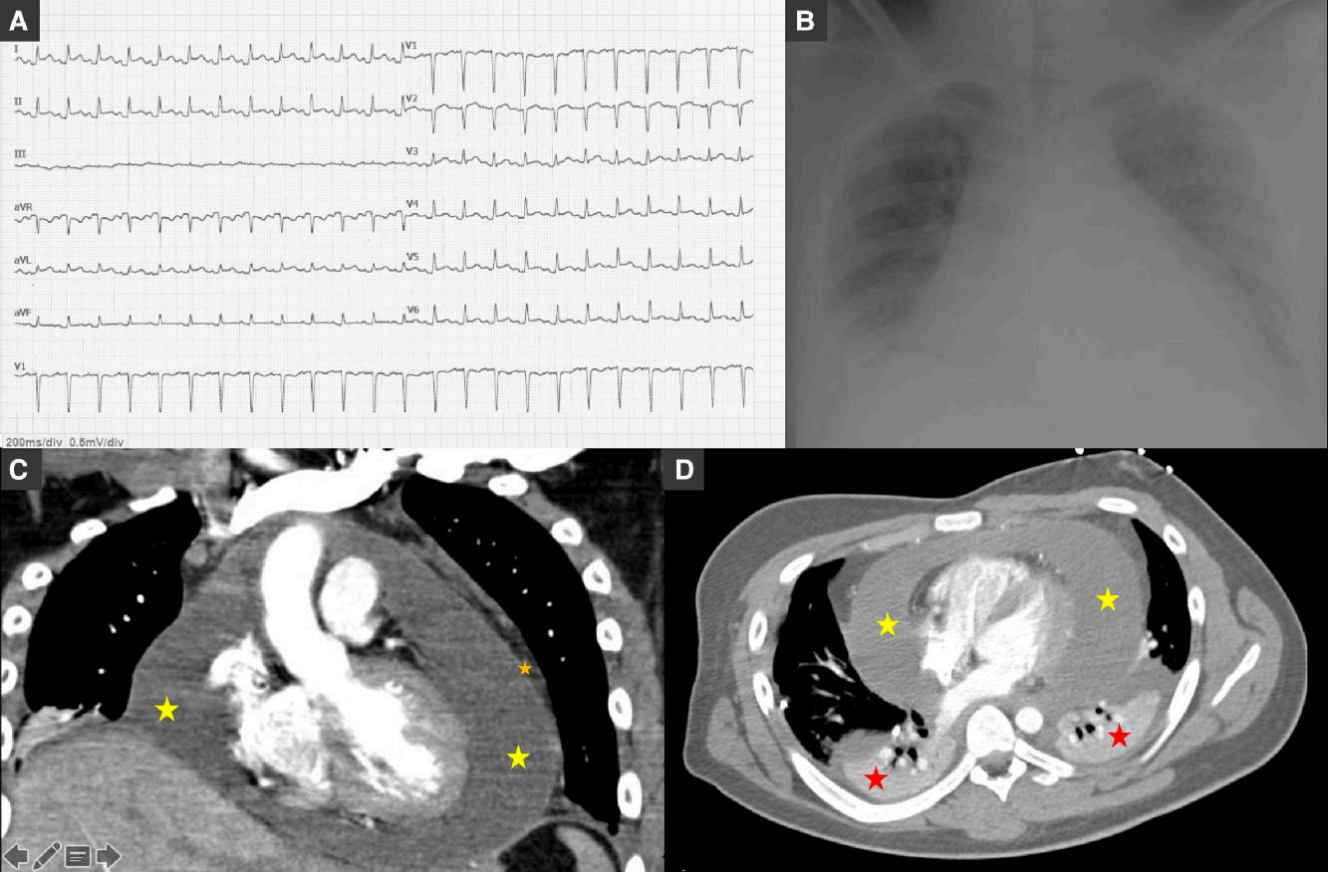
(*A*) Electrocardiogram showing sinus rhythm, normal PQ interval, QRS with normal duration, elevation of the ST-segment in leads V3–V6, aVL, LI, and LII, and ST-segment depression in aVR. (*B*) Chest radiography showing severe cardiomegaly, bilateral pleural effusion with large right-sided pleural effusion, parabronchial cuffing, and perihilar haziness. (*C* and *D*) Thorax computed tomography confirmed a circumferential pericardial effusion (yellow star) with maximum dimension of 40 mm with a thick pericardium (orange star) and bilateral pleural effusion (red star; with chest tube inserted in the right fifth intercostal space) in coronal (*C*) and axial (soft tissue) views (*D*).

We report the case of a 19-year-old male who presented to the emergency department with fever, chest pain, and dyspnoea for 2 days. On examination, he was febrile (39.5°C), normotensive, tachycardic (154 b.p.m.), tachypnoeic (22 b.p.m.), and hypoxemic (SpO_2_ 93% room air). Head, eyes, nose, throat, and neck examinations were unremarkable. Breath sounds were decreased in the bases, and fine crackles were noted. Auscultation of the heart revealed muffled heart sounds. The abdomen was soft and non-tender. The extremities were well perfused with strong and symmetric pulses. No oedema or jugular venous distention was appreciated. Electrocardiogram (ECG) showed depression of the PQ interval in aVF, LI, and LII, elevation of the ST-segment in leads V4–V6, aVL, LI, and LII, and ST segment depression in aVR (*Figure 1A*). Arterial blood gas showed mild hypoxaemia pO_2_ 63.8 mmHg and hyperlactacidaemia 2.9 mmol/L. Laboratory tests revealed normal haemoglobin 13.6 g/dL, leucocytosis 11.949/µL, neutrophilia 10.500/µL, lymphocytopaenia 890/µL, normal platelet count 135.000/µL, elevated C-reactive protein 30 mg/dL, elevated procalcitonin 0.48 ng/mL, N-terminal-proBNP of 632 pg/mL, and undetected troponin I. Renal function was normal. Chest radiography showed severe cardiomegaly, bilateral pleural effusion (more severe on the right), parabronchial cuffing, and perihilar haziness suggestive of congestion (*Figure 1B*). Echocardiogram showed normal size ventricles with preserved systolic function, valves with normal morphology and function, and a large circumferential pericardial effusion (22–25 mm) without collapse of the cardiac chambers or haemodynamical instability. Empirical antibiotic therapy with amoxicillin, loop diuretics, nonsteroidal anti-inflammatory drugs (NSAIDs, ibuprofen 600 mg t.i.d.), and colchicine (0.5 mg b.i.d.) was started. The patient was admitted to the intensive care unit (ICU), and a right thoracentesis was performed with insertion of a chest drain in the right fifth intercostal space (8–10 cm) and drainage of 600 cc of serosanguineous fluid. Pleural fluid culture was negative, and immunohistochemical analysis was compatible with exudate. A thorax computed tomography confirmed a circumferential pericardial effusion with maximum dimension of 40 mm (yellow star) with a thick pericardium and bilateral pleural effusion (with chest tube inserted in the right fifth intercostal space, red star; *Figure 1B* and *C*).

**Figure 2:**
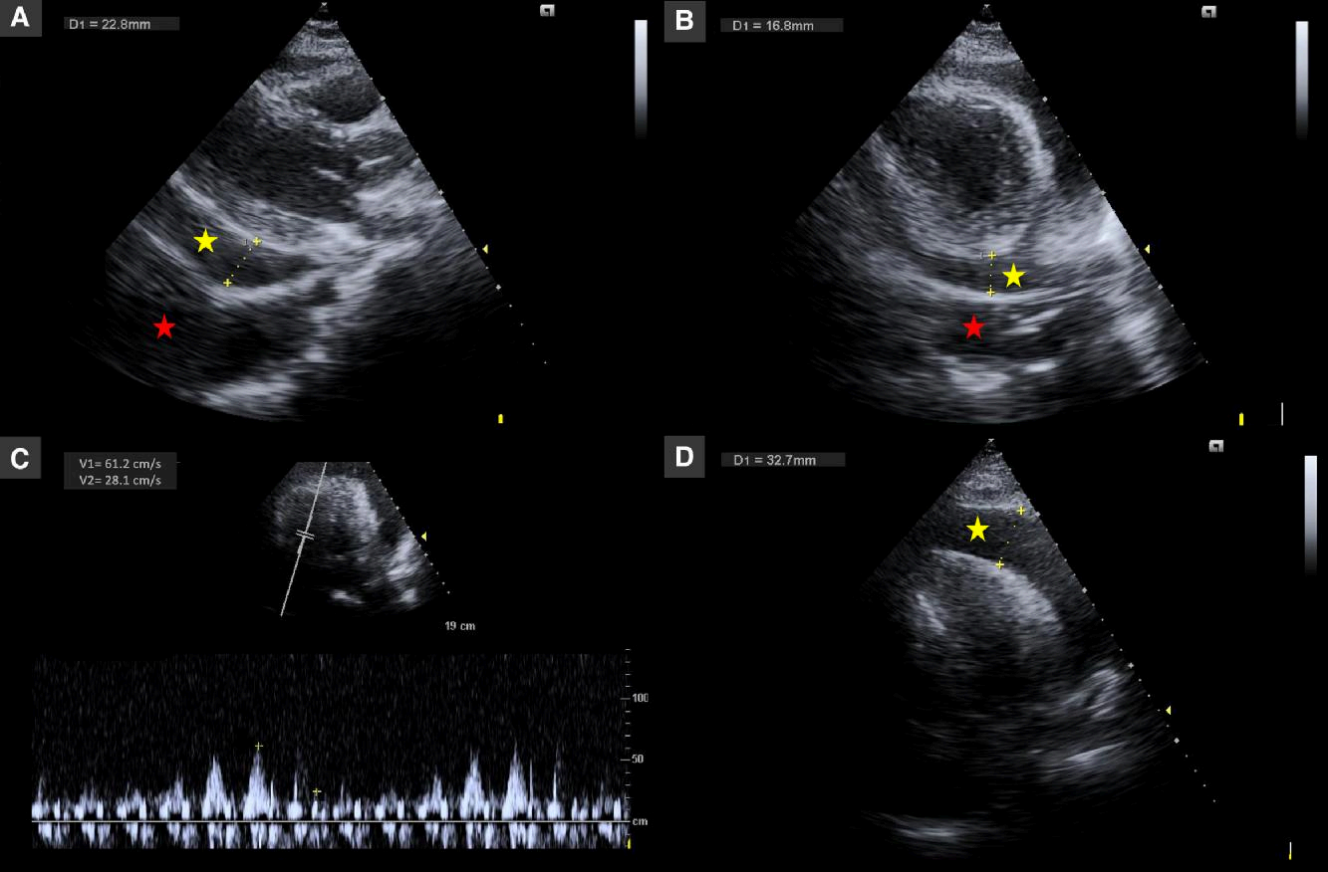
Echocardiography in parasternal long-axis (*A*), parasternal short-axis (*B*), and apical four-chamber (*C* and *D*) views showing pericardial effusion with large dimensions (30–33 mm, yellow star) with collapse of the right cavities and significant tricuspid inflow variation (46%) and pleural effusion (red star).

Two days later, the patient became haemodynamically unstable with hypotension (94/56 mmHg), and an echocardiogram confirmed enlargement of the pericardial effusion (red star) with collapse of the right cavities and significant tricuspid inflow variation (*Figure 2*). An urgent pericardiocentesis was performed with success. Immunohistochemical analysis was compatible with exudate with inflammatory cells. Autoimmunity laboratory tests were requested. Lupus anticoagulant and antinuclear antibody (ANA) with a speckled pattern were positive (1:160). Complement C4 was mildly reduced (7.6 mg/dL), and C3 level was normal. Anti-neutrophil cytoplasmic antibody, anti-mitochondrial antibodies, and anti-dsDNA were all negative. Interferon-gamma release assay, atypical and serology screening including herpesviruses, enterovirus, adenovirus, Epstein–Barr virus, coxsackie A and B, viral hepatitis, cytomegalovirus, human immunodeficiency virus, and *Aspergillus* were unremarkable. A score of 11 in the European League Against Rheumatism/American College of Rheumatology (EULAR/ACR) criteria for SLE was obtained confirming the diagnosis. Nonsteroidal anti-inflammatory drugs (ibuprofen 600 mg t.i.d.) and colchicine (0.5 mg b.i.d.) were continued, and high-dose corticosteroid [methylprednisolone 1 g/day during 2 days and then oral prednisolone 60 mg/day (1.0 mg/kg)] was started. The patient remained haemodynamically stable with normalization of the heart rate and resolution of the fever and pericardial effusion. The chest tube was removed after 3 days, and the patient was discharged after 7 days.

One month later, the patient returned to the emergency department with dyspnoea and a recurrence of fever, and a recurrence of polyserositis with small pleural and pericardial effusions without haemodynamic instability was confirmed. Loop diuretic and corticosteroid [oral prednisolone 90 mg/day (1.5 mg/kg)] were titrated. The patient improved without the need for thoracentesis or pericardiocentesis and was discharged after 4 days.

Corticosteroids and NSAIDs were tapered over 7 months and then suspended. Colchicine was maintained for 1 year and suspended afterwards without recurrence. To date, the patient remains asymptomatic, and follow-up echocardiogram 6 months later showed a very mild pericardial effusion without haemodynamic compromise or constriction.

## Discussion

Systemic lupus erythematosus is a chronic autoimmune disease characterized by multisystem inflammation. Cardiac involvement is common and often presents as acute pericarditis and may be present in up to 25% of the patients throughout the course of the disease.^1,2^ Pleuritis with pleural effusion is common and has been described in up to 30–50% of the cases, but it is usually small and rarely needs invasive procedures to drain.^2^ Pericardial effusion can be present in up to 2% of the patients, and significant pericardial effusion or cardiac tamponade is present in <1% of the cases of SLE and is even rarer as the first manifestation.^1^ One study reported incidence of up to 22% of tamponade but usually years after the diagnosis of SLE.^3^ Symptomatic polyserositis with pleural, pericardial, and/or ascites has been identified in 12–30% of the patients with SLE.^4,5^ Although SLE is more common in female patients, some studies have reported a higher incidence of pericardial serositis in male patients.^1^

The diagnosis of SLE encompasses a clinical challenge and requires a high clinical suspicion. In this case after positive ANA with a speckled pattern was found, the EULAR/ACR classification criteria were assessed, and a score of 11 points was obtained confirming the diagnosis (acute pericarditis, fever, and low complement C4 level).^6^ A positive ANA test is a nonspecific finding, but in this case, the presence of recurrent pericarditis with large pericardial effusion and the presence of other findings as lymphopaenia were red flags that raise the suspicion of an underlying autoimmune disease. No sign of peritonitis or ascites was identified as well as other typical signs of SLE as cutaneous, arthritis, renal, or neurological criteria.

The prognosis of polyserositis associated with SLE is usually good as long as it is identified and appropriate and attempted treatment is started.^4^ Relapse of serositis is rare and often responds to NSAIDs and corticosteroids, as happened in this case.^4^

This case report illustrates a rare form of presentation of SLE, in a young male, in which the first manifestation was recurrent pericarditis with polyserositis and cardiac tamponade.

## Lead author biography


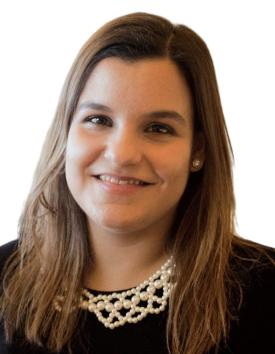
Dr Maria Inês Barradas completed her medical training at the University of Coimbra, and she is currently in her last year of cardiology training in Portugal. Her research interests include cardiac imaging, cardiomyopathies, and heart failure.

**Consent:** The authors confirm that written consent for submission and publication of this case report, including the images and associated text, has been obtained from the patient in line with COPE guidance.

**Funding:** Self-finance.

## Data Availability

The data underlying this article are available in the article.
